# DNA-based hydrogels: Ideal biomaterials for cartilage organoids

**DOI:** 10.1016/j.fmre.2024.04.001

**Published:** 2024-04-10

**Authors:** Congyi Shen, Zuhao Li, Guangfeng Li, Guangchao Wang, Zhen Geng, Jiacan Su

**Affiliations:** a Institute of Translational Medicine, Shanghai University, Shanghai 200444, China; b Organoid Research Center, Shanghai University, Shanghai 200444, China; c School of Medicine, Shanghai University, Shanghai 200444, China; d School of Life Sciences, Shanghai University, Shanghai 200444, China; e Department of Orthopedics, Shanghai Zhongye Hospital, Shanghai 200941, China; f National Center for Translational Medicine (Shanghai) SHU Branch, Shanghai University, Shanghai 200444, China; g Department of Orthopedics, Xinhua Hospital, Shanghai Jiao Tong University School of Medicine, Shanghai 200092, China

**Keywords:** DNA hydrogels, Tissue engineering, Organoid, Biomaterials, Cartilage repair

## Abstract

Osteoarthritis (OA) is a common degenerative disease with high disability rate, imposing significant economic burdens on individuals and society. Due to the limited self-repair ability of articular cartilage, the existing treatment methods still cannot effectively treat OA. Organoids are multicellular structures differentiated from stem cells or organ progenitors and can be used to model disease. Future applications can provide alternative organ replacement strategies. Therefore, constructing cartilage organoids is expected to overcome the shortcomings of the existing treatment methods to achieve effective treatment of OA. The construction of organoids requires three-dimensional network scaffolds resembling extracellular matrix (ECM) to support cell expansion. Hydrogel has a hydrophilic natural network structure. Hence it can mimic the ECM, providing mechanical support and a favorable microenvironment for cell growth. DNA hydrogel is mainly formed by DNA. It preserves DNA's programmability, biocompatibility and biodegradability, and has unique mechanical properties. Thus, it is an ideal material for constructing cartilage organoids. This review summarized the preparation methods and discussed the use of DNA hydrogels in the construction of cartilage organoids, aiming to provide a reference for the construction and design of cartilage organoids based on DNA hydrogels.

## Highlights

• Using cartilage organoids is expected to be an effective treatment of osteoarthritis.
• We first proposed to construct cartilage organoids by using DNA hydrogel.
• DNA hydrogel can replace Matrigel to construct cartilage organoids.
• DNA hydrogel has incomparable advantages of in constructing cartilage organoids.
• Using cartilage organoids to treat osteoarthritis has a great application prospect.

## Graphical abstract

**Figure:**
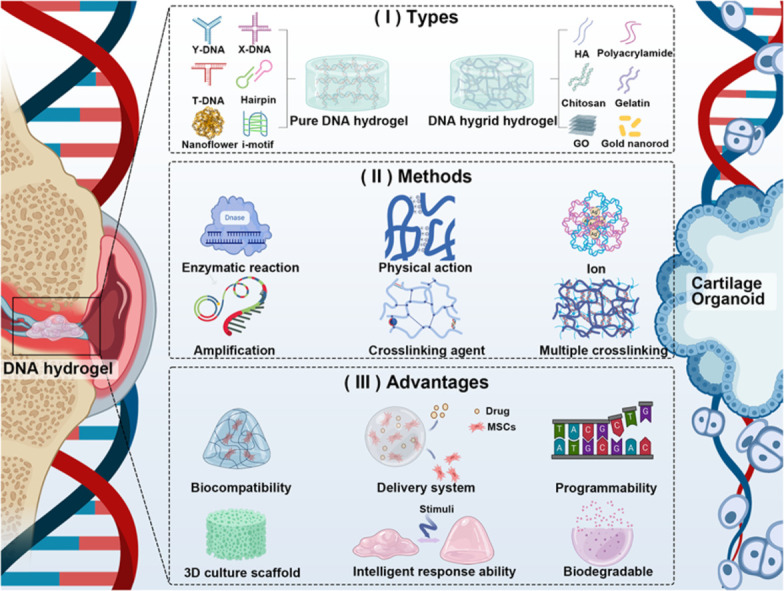

## 1 Introduction

Osteoarthritis (OA) is a common degenerative disease, and the most common form leading to disability in adults worldwide [1], [2], [3]. Age, gender, previous joint injury, obesity, genetic susceptibility and mechanical factors are common risk factors for OA. OA is expected to become one of the most prevalent diseases in high-income countries in the coming decades [4,5]. It is estimated that over 500 million people are affected worldwide, placing a huge burden on both individuals and socio-economics [6], [7], [8], [9], [10]. Previous studies showed that the pathological characteristics of OA were mainly due to ‘wear and tear’ or injury (including sports-related injuries) leading to the degeneration of articular cartilage [9,11]. Articular cartilage is the connective tissue that covers the surface of the articular epiphysis [12]. Because articular cartilage lacks blood vessels, nerves and lymphatic tissue, its self-repairing ability is limited [13]. Currently used strategies for cartilage defect repair include microfracture, osteochondral autograft transfer (OAT), osteochondral allograft transplantation and so on [11,^14^,15]. Despite their widespread clinical use, these methods still have some limitations and drawbacks [16]. For example, the complications of microfracture often include poor defect filling, osteophytes, infection and potential avascular necrosis [17]. Correspondingly, OAT is complicated by hemarthrosis, donor-site morbidity and donor-recipient site incongruity. Additionally, osteochondral allograft transplantation faces the challenge of mismatching the allograft's shape and the native knee structure, leading to biomechanical loading imbalances and subsequent degenerative joint changes. Furthermore, none of these methods solves the problem of long-term joint degeneration [18,19].

Currently, tissue engineering techniques based on cartilage regeneration have provided novel cartilage repair strategies, including autologous chondrocyte implantation (ACI) and matrix-induced autologous chondrocyte implantation (MACI) [12,20]. However, both ACI and MACI require two surgical procedures and the current chondrocyte expansion strategy relies heavily on the 2D system. This system not only requires a long time but also causes chondrocyte dedifferentiation, leading to the formation of fibrocartilage [21]. To improve new tissue formation, graft maturation and biomechanical integrity, it is necessary to culture cells in 3D *in vitro*. Cartilage is a heterogeneous tissue composed of layers with different functional and biochemical properties, and the repair effect with homogeneous implants is limited [22]. Therefore, the construction of cartilage organoids as *in vitro* 3D research models or graft materials for cartilage regeneration is expected to provide new ideas for cartilage tissue engineering [23], [24], [25]. The construction of cartilage organoids requires 3D network scaffolds similar to cartilage extracellular matrix (ECM) to support the expansion of chondrocytes. Hydrogels have hydrophilic natural reticular structures with advantages such as biocompatibility and biodegradability [26], [27], [28]. Hence, they can mimic the ECM to provide mechanical support and a favorable microenvironment for cell growth, thereby simulating the physiological niche of chondrocytes.

In recent years, DNA hydrogels, with their unique nucleic acid-based features, have garnered interest. DNA, essential for the growth and development of most organisms, encodes, stores, and transmits genetic information through the principle of base pairing. The powerful assembly capacity of DNA is conferred by the highly selective recognition of these bases and the programmability of the sequence code [29]. Thus, DNA has the advantages of programmability, easy modification, predictable secondary structure, biocompatibility and biodegradability, which shows great potential in the construction of biological materials [30,31]. DNA hydrogels are 3D mesh polymeric materials formed mainly with the participation of DNA. And their applications have progressively expanded from the field of life sciences to the biomedical field due to the perfect combination of the biological properties of the retained DNA and the mechanical properties of its skeleton [32], [33], [34]. In addition, DNA-based biomaterials have incomparable advantages over other materials in cartilage repair: (1) Good biocompatibility (DNA is a natural biological macromolecule); (2) DNA hydrogel can be specifically degraded by endonuclease, exonuclease, and so on; (3) programmability (nucleic acids with certain sequences can be designed and synthesized by solid phase synthesis and molecular biology techniques); (4) controlled phase transition (some functional DNA will undergo a sudden conformational change after external stimulation, which will bring reversible and switchable changes to DNA hydrogel); (5) adjustable mechanical properties; (6) accessibility (DNA sequence can be rapidly prepared or amplified by modern DNA synthesis technology such as automation technology, polymerase chain reaction (PCR) or production in microorganisms) [35], [36], [37], [38], [39], [40], [41]. Therefore, DNA hydrogel holds considerable potential for research and application in cartilage repair. Moreover, DNA hydrogel is a potential outstanding material for cartilage organoids construction.

Herein, we reviewed the research progress in the preparation methods and practical applications of DNA hydrogels and discussed the future research direction and application prospect of DNA hydrogels in cartilage repairing. Overall, our aim is to use DNA hydrogel to construct cartilage organoids. Cartilage organoids will serve not only as *in vitro* models for disease and defect studies, including mechanisms of disease progression and cartilage repair, but also for drug screening and action analysis. Furthermore, they are envisioned as innovative implants for cartilage repair, potentially supplanting autologous cartilage transplantation.

## 2 Composition of DNA hydrogels

About thirty years ago, DNA molecule, as a promising nanomaterial, received extensive attention due to its accurate molecular recognition ability, programmable sequence and predictable secondary structure [42]. It has been widely used to prepare 2D and 3D nanostructures of specified sizes with precise addressability [43], [44], [45], [46]. But it is not used to prepare bulk materials.

In 1996, Nagahara and Matsuda first reported the preparation of temperature-responsive hydrogels by DNA modification of the side chains of water-soluble synthetic polymers to form a single crosslinking point [47]. Since then, DNA hydrogel has come into our sight. Now, DNA nanotechnology improves by leaps and bounds. DNA has become an outstanding construction material for hydrogel [48,49]. It is well known that DNA consists of nucleobases and sugar-phosphate backbone. There are four nucleobases: adenine (A), guanine (G), thymine (T) and cytosine (C). In terms of composition, DNA hydrogels can be mainly divided into two types, namely, pure DNA hydrogels and DNA hybrid hydrogels. Pure DNA hydrogels utilize DNA as the sole component, formed through chemical actions (with chemical bonds as crosslinking points) or physical interactions (non-covalent actions like hydrogen bonding, van der Waals forces, or intermolecular entanglement of DNA strands). These hydrogels have precise structural control, specific reactivity, and good biodegradability. In 2006, Luo et al. first reported pure DNA hydrogels constructed only by DNA branches mediated by ligases [50]. In 2009, Liu et al. first constructed the self-assembled DNA hydrogel which consists of only three single-stranded DNA (ssDNA) [51]. In 2012, Li et al. first designed DNA hydrogels self-assembled by physical action (non-covalent bond) through long-chain DNA [52]. Notably, the excellent thixotropy of this DNA hydrogel allows it to be easily injected through the injector [53]. In the context of pure DNA hydrogels, DNA supramolecular hydrogels merit special mention. Viewed as a specific subclass of DNA hydrogels, they are founded on a polymeric network constituted by DNA molecules. DNA supramolecular hydrogels emerge from self-assembling structures brought into being through non-covalent interactions, including hydrogen bonds, hydrophobic forces, and ionic bonds. Within these hydrogels, DNA molecules spontaneously organize into larger formations via these weaker interactions, intertwining and cross-linking to cultivate a stable three-dimensional network. Distinguished from other DNA hydrogels, supramolecular hydrogels are characterized by their self-assembly properties and the dynamic reversibility of their formation. However, the modification of DNA-polymers requires multiple steps and the development of pure DNA hydrogels is limited to some extent.

For DNA hybrid hydrogels, DNA mainly played a crosslinking role during the gelling process. DNA is widely used as a crosslinking agent in the preparation of hydrogels because it can be simply attached to the skeleton as a side chain. At present, the reported skeleton materials are hyaluronic acid (HA), polyacrylamide, chitosan, gelatin and so on [54], [55], [56], [57], [58], [59], [60], [61]. The designed DNA side chains can impart different responsiveness to the hydrogel, such as temperature, wavelength, pH, stiffness, and small signal molecule [41,^47^,[61], [62], [63], [64], [65], [66], [67]. Although the synthesis cost of oligonucleotides is decreasing (its effective cost is halved every 30 months), the preparation cost of DNA-based hydrogels is still very high [68]. The development of the hybrid hydrogel can solve the problem of high cost and is beneficial to the large-scale preparation of the functional nucleic acid DNA hydrogel. Aptamers are short single-stranded oligonucleotide molecules (RNA or DNA) that can be folded into complex three-dimensional structures, enabling them to specifically recognize targets from small ions to the entire organism [69]. Conjugating aptamers with electrochemical indicators and nanoparticles into diverse structural units, or incorporating single or multiple DNA restriction enzyme sites into hydrogels through sequence design, can markedly broaden their responsive scope [70], [71], [72], [73], [74]. In contrast, DNA hybrid hydrogels have strong mechanical properties and can be easily changed by sequence design.

The following section discusses the preparation methods of various DNA-based hydrogels.

## 3 Preparing methods of DNA hydrogel

Based on the formation mechanisms, DNA hydrogels can be categorized into chemical gels and physical gels. Physical gels are primarily formed through non-covalent interactions such as hydrogen bonding or electrostatic interactions, along with the entanglement of DNA strands. Chemical gels, on the other hand, can be synthesized by modifying hydrophilic polymer chains with DNA as side chains, followed by crosslinking these side chains to form DNA hydrogels, or by using DNA ligases to covalently link phosphodiester bonds, thereby crosslinking the DNA itself to form the hydrogel. There are numerous methods for preparing DNA hydrogels. And the advantages and limitations of the above methods are presented in Table 1.

**Table 1:** **Methods for synthesizing DNA hydrogels**.

DNA hydrogel type	Methods	Advantages	Limitations
Pure DNA hydrogels	DNA nanostructure units	Rapid formation and reversibility; precision and control	Randomness in pore spacing; limited scalability
	Nucleic acid amplification technique	High programmability; cost-effectiveness; stable and efficient	Time-consuming processes; dependency on specific conditions
DNA hybrid hydrogels	Physical action	Self-healing; biocompatibility and luminescent characteristics	Complexity in kinetic adjustment; potential instability
	Crosslinking agent	Strong and stable bonds; controlled release	Potential toxicity
	Ion	Tunable properties; conformational maintenance and functionality	Potential for metal ion toxicity; dependence on metal ion concentration and pH

### 3.1 Preparation of pure DNA hydrogel

#### 3.1.1 DNA nanostructure units

The preparation of pure DNA hydrogels using nanostructured units is the most common method. T4 DNA ligase is mainly used to synthesize DNA nanostructure units.

**Fig. 1:**
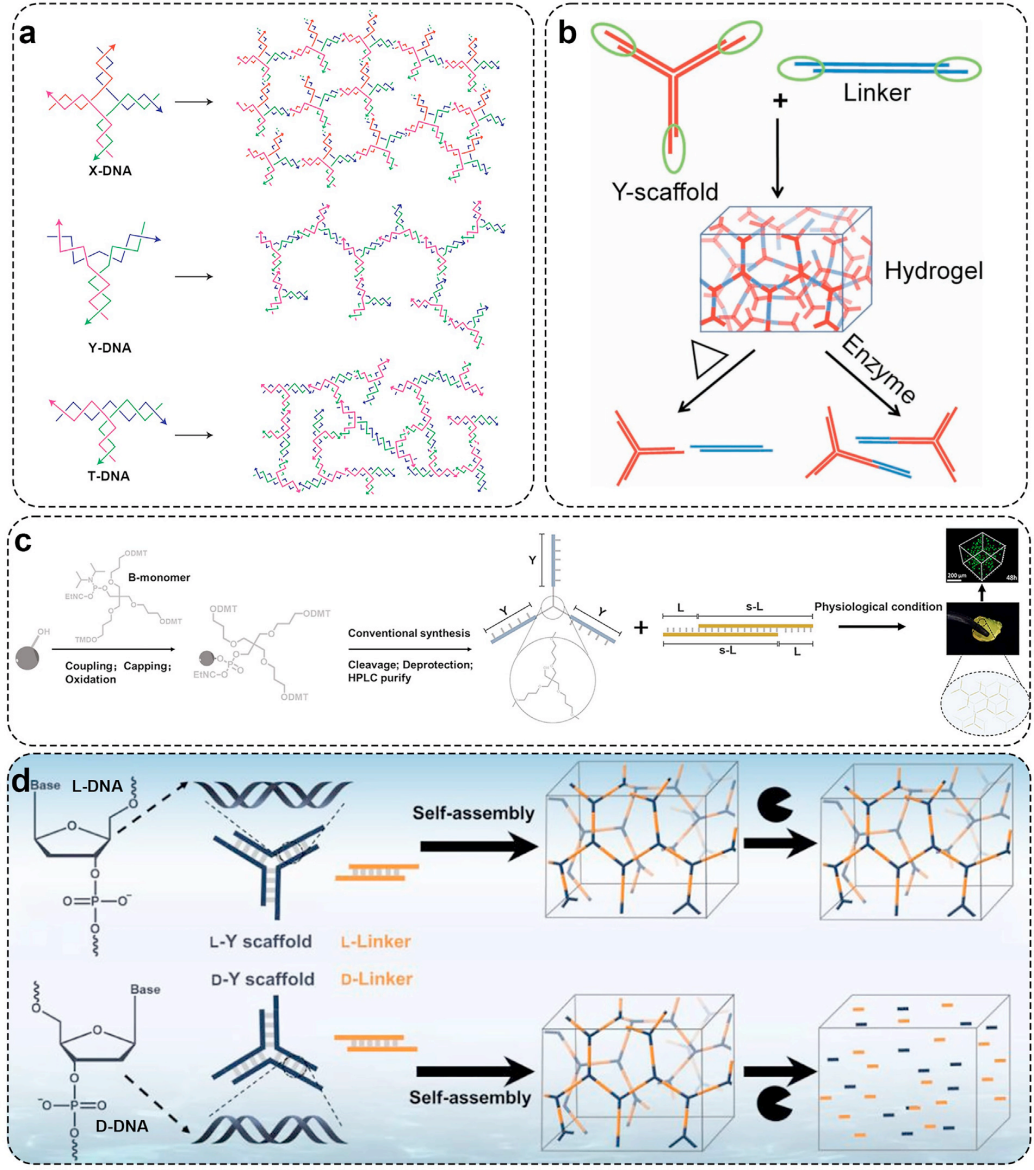
**Preparation of pure DNA hydrogel using DNA nanostructure units.** (a) Three common DNA nanostructure units for hydrogel preparation synthesized by T4 DNA ligase. Adapted with permission from [50], copyright 2007, Nature Materials. (b) The Y-scaffolds and the linkers form DNA hydrogel with reversible thermal stimulation response and enzyme response. Adapted with permission from [75], copyright 2011, Advanced Materials. (c) Diagram of synthesis process of B-monomers and the formation process of the DNA pure hydrogel. Adapted with permission from [79], copyright 2021, ACS Applied Materials & Interfaces. (d) Schematic diagram of D-DNA and L-DNA hydrogel synthesis. Adapted with permission from [80], copyright 2022, Angewandte Chemie-International Edition.

Currently, the types of units for constructing DNA hydrogels include X-type, T-type, and Y-type structures. As aforementioned, Um *et al*. first reported a method for constructing DNA hydrogels entirely through covalent phosphodiester bonds catalyzed by T4 DNA ligase linking DNA units in 2006 [50]. The DNA hydrogel formed by respectively assembling three structural units with different shapes (X-type, T-type and Y-type) has degradability and good biocompatibility (Fig. 1a). The pore size of the hydrogel constructed by DNA nano-units can be accurately controlled. Compared with the X-DNA hydrogel with regular pore spacing, the pore spacing of the gel formed by Y-DNA or T-DNA is more random. Due to the lengthy duration required for enzyme linking, researchers have further refined the process of forming hydrogels from DNA nanostructure units, utilizing the inherent base pairing of DNA to prepare physical DNA hydrogels. For example, Xing *et al*. designed a Y-type building unit containing three ‘sticky ends’ and simultaneously introduced a linear DNA double-strand with two ends containing ‘sticky ends’ as a ‘linker’ to form hydrogel through hybridization between the Y-type building block and the ‘sticky ends’ of the linker (Fig. 1b) [75]. The prepared DNA hydrogel formed rapidly (within 1 min) without any chemical treatment and had reversible thermal stimulation response and enzyme response. In subsequent research reports, researchers have found that hydrogels can be enhanced, softened, and even fluidized by lengthening and shortening the lengths of DNA linkers or by inserting into mismatch sites [76]. A stimulus-responsive DNA hydrogel can be prepared by introducing a functional nucleic acid sequence into a viscous terminal sequence or linker of a DNA structural unit [51,^77^,78]. Recently, studies have reported that Y-shaped DNA can be directly coupled using B monomers to form structures. Moreover, this Y-shaped DNA shows efficient assembly behavior and forms thermal stable assembly (Fig. 1c) [79]. Interestingly, Liu *et al*. reported a hydrogel composed of L-DNA, which exhibits enhanced stability against nucleases compared to D-DNA hydrogels, resulting in superior gel stability (Fig. 1d) [80].

#### 3.1.2 Nucleic acid amplification technique

Since the formation of DNA building blocks requires the participation of a large number of chemically synthesized deoxyribonucleotides, its high manufacturing cost limits the practical application of DNA hydrogels. By contrast, nucleic acid amplification technology, which achieves rapid amplification with low error rate and good stability, has been gradually applied to the preparation of DNA hydrogels. By introducing more constant temperature amplification methods, the researchers reduced the dependence of the amplification process on variable temperature instruments.

**Fig. 2:**
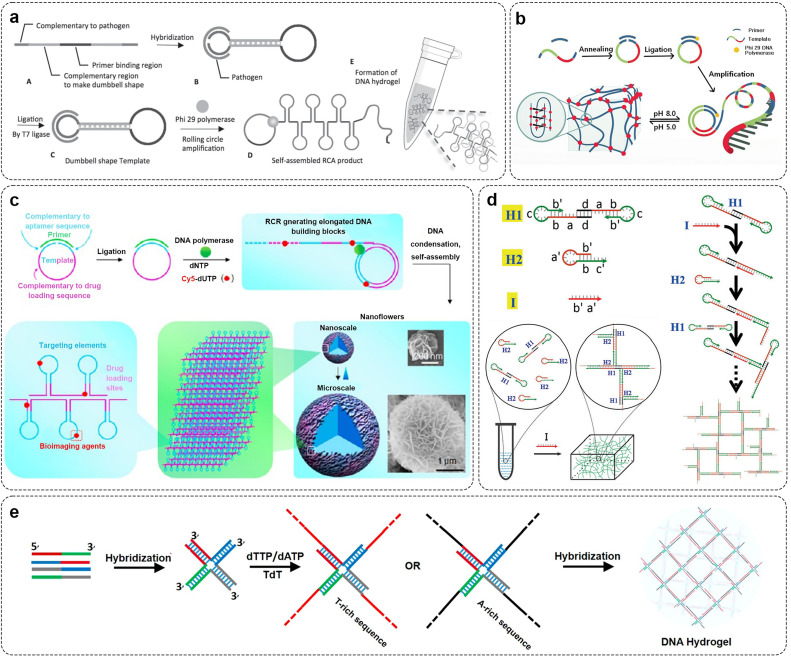
**Preparation of DNA pure hydrogel by modern nucleic acid amplification technology.** (a) DNA hydrogel with dumbbell-shaped sequence formed by RCA technology. Adapted with permission from [82], copyright 2017, Advanced Materials. (b) Schematic diagram of DNA hydrogel containing i-motif sequence formed by RCA technology. Adapted with permission from [83], copyright 2017, Chemistry-A European Journal. (c) DNA nanoflower structures with RCA participation. Adapted with permission from [88], copyright 2013, Journal of the American Chemical Society. (d) Mechanism of DNA hydrogel prepared by C-HCR. Adapted with permission from [91], copyright 2017, Angewandte Chemie-International Edition. (e) TdT participates in hydrogel formation. Adapted with permission from [95], copyright 2016, ACS Applied Materials & Interfaces.

Rolling circle amplification (RCA) is the synthesis of ultra-long ssDNA molecules with periodic repetitive sequences using ssDNA as the template by Φ29 DNA polymerase under the isothermal (30℃) condition [81]. Its advantages include mild reaction conditions and stability and efficiency in complex biological environments. Due to the flexibility of ssDNA molecules and the fact that DNA molecules are easy to form specific spatial structures, it is easy to construct DNA hydrogel frameworks by physical entanglement of long-stranded DNA molecules. By designing the complementary sequences of various functional nucleic acid sequences into the cyclic template, the RCA hydrogel can be endowed with the corresponding functions. For example, Li *et al*. introduced pathogen binding sites into molecular padlock templates (Fig. 2a) [82]. After hybridization to the target pathogen sequence, the self-assembled regions may be ligated to form a closed circular template for the RCA process. Complementary single-strand DNA products generate by that RCA process can form self-assembled dumbbell-shaped multiple tandem repeats along the linear DNA chain which facilitate physical entanglement of the DNA chain to form DNA hydrogels. Complementary single-stranded DNA products generated through the RCA process can form self-assembled dumbbells along a linear DNA chain with multiple tandem repeat sequences, facilitating physical entanglement of DNA strands to form DNA hydrogels. According to the nature of the i-motif forming sequence, i-motif structures can be formed under acidic conditions. As shown in Fig. 2b, Xu et al. leveraged the property of i-motif forming sequences to adopt i-motif structures under acidic conditions. Utilizing RCA technology to amplify units containing i-motif sequences, they successfully prepared pH-responsive hydrogels via the formation of intermolecular/intramolecular i-motifs [83]. Similarly, Zhu *et al*. used this pH-responsive i-motif structure to prepare DNA hydrogels that can achieve smart intracellular release of mRNA [84]. Additionally, Huang *et al*. introduced intermolecular G-quadruplex into the RCA products and crosslinked to form hydrogels. Simultaneously, the G-quadruplex combined with heme to form DNA mimic enzyme with catalase activity [85]. Notably, DNA hydrogels formed with the participation of RCA could observe nanoflower structures with uniform size on the micro-scale (Fig. 2c). These nanoflower structures were self-assembled from DNA and magnesium pyrophosphate, a by-product of DNA polymerization. And that structural integrity of the nanoflower structure can still be maintained under the treatment of nuclease, human serum, high temperature, urea or dilution [86], [87], [88]. Researchers have also confirmed that the size and density of nanoflowers can be achieved by sequence design and controlling the enzyme reaction [52,89].

Hybridization chain reaction (HCR) is an isothermal nucleic acid amplification technique without enzyme participation. When the DNA hairpin probe encounters the initiator DNA strand, the two metastable DNA hairpin probes driven by the free energy form a long linear DNA duplex through a chain reaction of alternating hybridization [90]. Based on the property that the average size of HCR products is inversely proportional to the initiator concentration, the DNA hydrogel formed with the participation of HCR can be quantitatively regulated by designing DNA chain sequences, reaction concentration, and so on. As shown in Fig. 2d, Fan *et al*. developed a novel clamped hybridization chain reaction (C-HCR) [91]. In this method, a DNA hairpin probe creates a dimer (H1) by hybridizing its palindromic sections. This allows polymerization from both ends, transitioning from one-dimensional linear HCR products to a three-dimensional crosslinked network. By adding DNA initiator strands (I) at specific times, stable DNA hydrogels can be formed through non-enzymatic DNA hybridization, enabling precise control over space and time during gel formation. Based on the C-HCR, Li *et al*. further endowed the gelling behavior with pH responsiveness [92]. By introducing a segment of DNA which can hybridize with an initiating chain of the C-HCR under a neutral condition to form a pH-responsive molecular switch, the DNA forms a C-G·C+ three-chain structure under an acidic condition to generate a sol-gel transition of an initiating chain release triggering system of the C-HCR.

Terminal deoxynucleotidyl transferase (TdT) can catalyze the 3′-OH end polymerization of deoxyribonucleotides to extend DNA molecules of dsDNA (double-stranded DNA) or ssDNA with more than 3 nt deoxyribonucleotides in the absence of templates [93,94]. Fig. 2e shows that Xiang *et al*. extended 3′ hydroxyl end in the X-type DNA unit with a single base (dATP or dTTP) by TdT amplification technique to obtain two building blocks (X-DNA-An and X-DNA-Tn). The hydrogel was formed by complementary hybridization of the polyA tail and polyT tail of the building block. The participation of TdT decreased the required amount of primary DNA. Meanwhile, the hybridization-mediated crosslinking of the building blocks endowed the hydrogel with enhanced mechanical strength. [95]. Gao *et al*. used TdT technology to extend the chain at the vertex of the tetrahedron, and obtained two kinds of tetrahedrons containing polyA and polyT, respectively [96]. The amplified products of TdT were crosslinked by the Watson-Crick base pairing principle to form DNA hydrogel. T4 DNA ligase is also mainly used to synthesize DNA nanostructure units.

### 3.2 Preparation of DNA hybrid hydrogel

#### 3.2.1 Physical action

The phosphate groups in DNA strands confer an overall negative charge to DNA. Therefore, DNA and positively charged molecules could be crosslinked through strong electrostatic action to synthesize DNA hybrid hydrogel. In 2007, the first DNA hybrid hydrogel crosslinked by electrostatic interaction was prepared by mixing two DNA strands (dsDNA and ssDNA) with cetyltrimethylammonium bromide (CTAB) or lysozyme [97]. This study found that ssDNA interacted more strongly with the amphiphiles than dsDNA, indicating that the hydrophobic interaction also played an important part in the crosslinking of DNA hydrogels. Paul *et al*. created additional physical network crosslinking points based on the anisotropic charge distribution of two-dimensional silicate nanodisks (nSi) and the electrostatic interaction with DNA skeleton to enhance the mechanical elasticity of hydrogels [98]. Besides, Song *et al*. stably assembled hydrogels by binding AuNRs with X-shaped DNA building blocks through electrostatic interactions. [99]. What's more, Luo et al. reported a case of a hydrogel in which nucleic acid was electrostatically adsorbed by clay, and the adsorption rate to DNA was as high as 100% [100]. In this hydrogel, nucleic acids are not only protected from nucleases but also show enhanced transcription and translation reactions. It is speculated that early life evolution may occur in the clay hydrogel environment. π-π stacking widely exists in DNA double helix. Shi et al. designed a self-assembled hydrogel that adsorptively crosslinked graphene oxide (GO) and ssDNA chains formed *in situ* with π-π stacking and hydrophobic interaction [101]. Since the hydrogel is crosslinked in a non-covalent form, the hydrogel has strong self-healing property and environmental stability and simultaneously inherits excellent mechanical properties and dye adsorption capacity of graphene. Although the π -π interaction between GO and ssDNA was stronger than that with dsDNA, KURAPATI *et al*. prepared the first GO/dsDNA self-assembled hydrogel in aqueous buffer medium by changing the concentration of components [102]. This hydrogel exhibits strong binding between dsDNA and graphene, with tunable porosity achievable through concentration adjustments. Liu et al. designed a linear DNA sequence containing i-motif tail and wrapped SWNT with the strong π-π stacking between DNA and SWNT to form SWNT-DNA conjugate [66]. The sol-gel transition was achieved by adjusting the pH to control the formation of i-motif between the linear units.

#### 3.2.2 Crosslinking agent

According to the different crosslinking forms of different crosslinking agents, the crosslinking agents used for crosslinking DNA hybrid hydrogels are divided into two types: non-covalent crosslinking agents and covalent crosslinking agents.

**Fig. 3:**
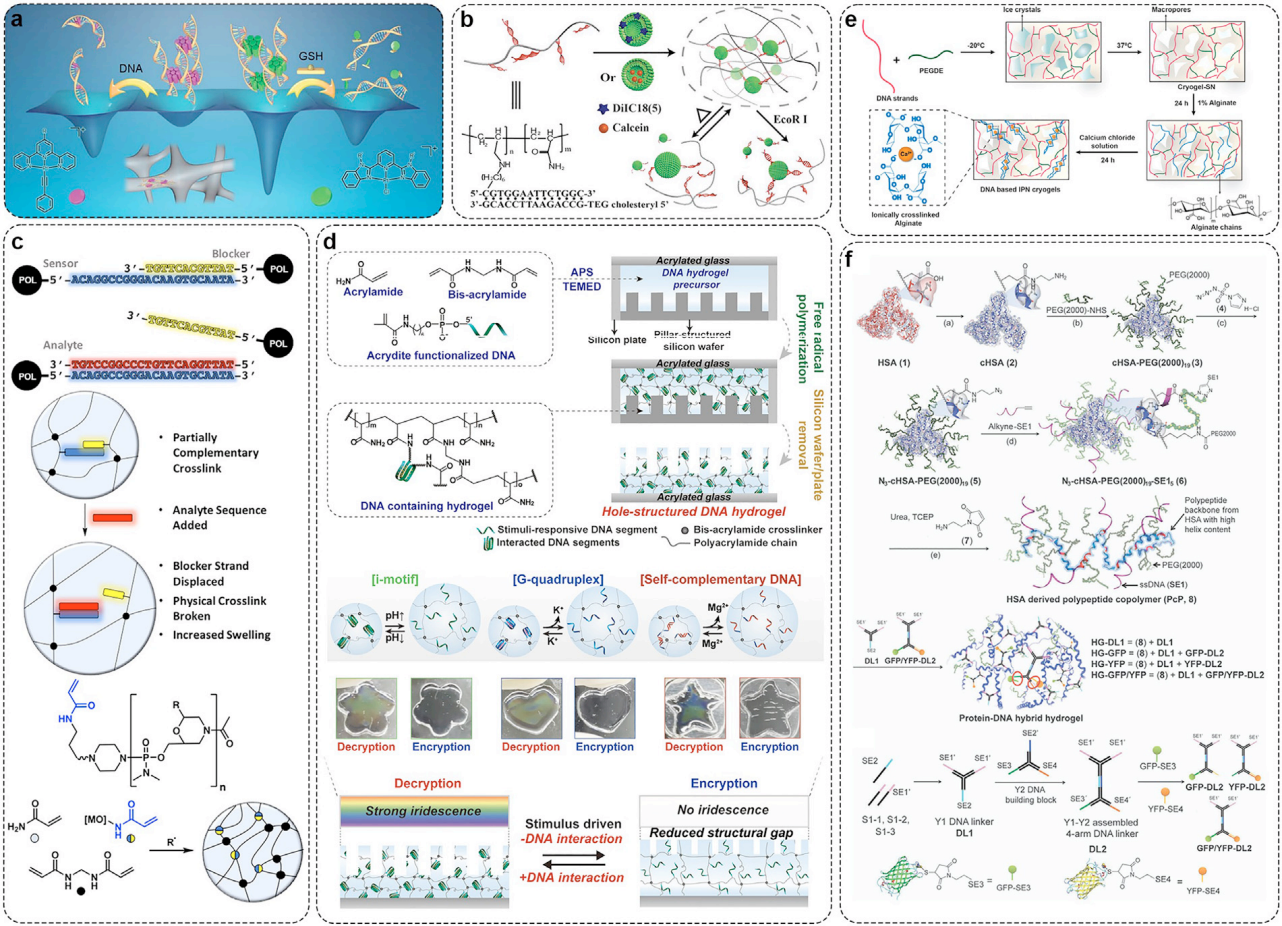
**Preparation of DNA hybrid hydrogel with crosslinking agent.** (a) Schematic diagram of platinum (II) non-covalent crosslinkers for the DNA hydrogel. Adapted with permission from [103], copyright 2020, Chemical Science. (b) Using liposome as noncovalent crosslinking agent to crosslink DNA and the theory of stimuli-responsive release system. Adapted with permission from [57], copyright 2018, Small. (c) Morpholino oligonucleotide crosslinked hydrogel system and its crosslinking principle. Adapted with permission from [105], copyright 2019, ACS Sensors. (d) Synthesis of DNA hybrid hydrogel from linked DNA and PEG by click chemistry and the schematic diagram of structure transformation principle. Adapted with permission from [106], copyright 2022, Nano Letters. (e) Preparation of hydrogel with PEGDE. Adapted with permission from [113], copyright 2020, ACS Macro Letters. (f) Protein and DNA connection and hydrogel synthesis by using click chemistry. Adapted with permission from [114], copyright 2014, Chemical Communications.

Non-covalent crosslinkers crosslink DNA with polymers by physical actions, such as electrostatic and hydrophobic interactions. Zhang *et al*. reported a case of hydrogel crosslinking smDNA with a platinum (II) complex as an electrostatic crosslinker (Fig. 3a) [103]. The platinum (II) complex was first accumulated into a columnar phase and then the DNA chain was crosslinked by electrostatic adsorption. If the DNA of the system is in a supersaturated state, the insertion of platinum (II) into the DNA base pairs will depolymerize the platinum (II) complex formed by the non-covalent metal-metal and π-π stacking, and a gel-sol transition will occur. The hydrogel inherits the luminescent characteristics of the platinum (II) polypyridine complex and the biocompatibility of DNA. Besides, the kinetic characteristics can be adjusted by changing the structure of the platinum (II) complex. Guo et al. reported an injectable polyacrylamide/DNA hydrogel crosslinked with liposomes as a non-covalent crosslinker based on the hydrophobic interaction between cholesterol groups and lipid bilayers (Fig. 3b) [57]. In this process, ssDNA is bound to acrylamide by free radical polymerization. DNA hydrogels crosslinked with non-covalent crosslinkers are self-healing. DNA can also be used as a crosslinking agent. Morpholino oligonucleotides (MOs), neutral DNA analogs, are functionalized with acrylamide groups. These MO-crosslinked hydrogels include both physical (hydrogen-bonded MOs) and covalent (MBA) crosslinked types. At present, MO has been reported as a physical crosslinking agent for the preparation of DNA-functionalized hydrogels for the visual detection of Hg^2+^ and adenosine [104]. A representative study shown in Fig. 3c prepared a DNA optical hydrogel by double bond polymerization using acrylamide functionalized MO [105]. Additionally, Kim *et al*. used DNA as a reversible cross-linking agent and PH and ions as stimuli to achieve structural color conversion (Fig. 3d) [106]. Representative studies also include oligonucleotide and convertible polyacrylamide hydrogels using DNA aptamers as crosslinkers and acrylamide-modified oligonucleotides as scaffolds [107].

Covalent crosslinking agents facilitate the formation of covalent bonds between DNA and the polymer. Ethylene glycol diglycidyl ether (EGDE) is a less toxic dialdehyde, capable of undergoing epoxide ring-opening reactions with hydroxyl, carboxyl, amino, and thiol groups. Therefore, EGDE has been widely used for crosslinking biopolymers [108]. Okay et al. studied the formation of DNA hydrogels catalyzed by N,N,N’,N’-Tetramethylethylenediamine (TEMED) under different conditions using EGDE crosslinking agent, and also studied its elastic modulus and expansion behavior [109,110]. Later, Costa et al. also studied the release mechanism of such hydrogels [111]. Queiroz et al. reported that DNA nano-hydrogels for controlled release used EGDE as a crosslinking agent and conjugated with polyamines to promote pDNA coagulation[112]. In addition to EGDE, polyethylene glycol diethylene oxide (PEGDE) is also a crosslinking agent for crosslinking biopolymers based on amine-epoxidation reactions. Paul et al. used PEGDE as the crosslinking agent to prepare DNA/alginate gel (Fig. 3e) [113]. They first crosslinked DNA and PEGDE at low temperature to form macroporous DNA scaffolds, subsequently integrating alginic acid chains to generate a network through low-temperature gel permeation. This interpenetrating network structure has the advantages of high hardness, excellent toughness and other advantages that a single network structure does not have, and has grand potential in tissue engineering applications. What's more, DNA can also be crosslinked with macromolecules by click chemistry. The cycloaddition reaction of alkyne and azide is the most used one. Weil et al. performed PEG modification on the plasma protein human serum albumin, subsequently modifying ssDNA with azide to facilitate its conjugation to PEG. Protein-DNA hybrid hydrogels are formed using conjugation between DNA (Fig. 3f) [114]. Ignatius et al. utilized a similar method to prepare DNA-protein hybrid hydrogels for the controlled release of DNA-tagged cargo, applicable in treating bone diseases [115].

#### 3.2.3 Ion

Metal ions play an essential role in the conformational maintenance, biosynthesis, function exertion and regulation of nucleic acids. The coordination between atoms or ions and ligand molecules can also form multiple groups of DNA hydrogels. The common examples are the long ssDNA molecules rich in T and C. T-Hg^2+^-T and C-Ag^+^-C metal-base pairs were formed under the driving of Hg^2+^ and Ag^+^. Finally, intramolecular fold occurs to form a special structure. Li et al. amplified long ssDNA molecules rich in C using RCA technology [116]. Hydrogels were formed by crosslinking on C-rich sequences through the interaction of silver nanoclusters and Cs. In addition, Willner et al. reported DNA hydrogels which consist of Y-scaffolds and nucleic acid-functionalized acrylamide chains. A convertible gel-to-solution transition was achieved by the C-Ag^+^-C complex formed by Ag^+^ stimulation [117]. Similarly, Hao et al. obtained DNA supramolecular hydrogels using 5′-adenosine monophosphate (AMP) and Ag^+^ [118]. The research found that the gelling ability of the hydrogels was enhanced by raising the concentration of Ag^+^ or reducing the pH. Geng et al. synthesized a complex based on Zn^2+^ with different counter-anions [119]. This Zn^2+^ complex could decompress dsDNA into ssDNA to enhance the interaction between the molecules of the assembled Zn^2+^ complex and form a three-dimensional matrix hydrogel using DNA as a crosslinking agent. The counterions, featuring terpyridine and glycosyl units connected by ethylenediamine, facilitate intercalation into ds-DNA through coordination and π-π stacking, enabling intermolecular hydrogen bonds for the self-assembly into three-dimensional matrix gels. Different gelation capabilities can be achieved by employing varied counterions. Thus, different gelling abilities can be adjusted by using a counter anion. Yang et al. synthesized a series of DNA supramolecular hydrogels by using the coordination and electrostatic interaction between lanthanide ions (Tb^3+^ and Eu^3+^) and linear ssDNA [120]. By adjusting the Tb/Eu ratio, DNA supramolecular hydrogels exhibited a green-to-yellow tunable luminescence and reversible luminescence-stimulating reactivity to Ag^+^/L-Cys. This research is expected to promote the application development of supramolecular hydrogels in optical display materials and small molecule sensors.

#### 3.2.4 Multiple crosslinking

The synthesis of DNA hybrid hydrogels can combine multiple interactions or crosslinking methods, not just a single approach, to better regulate the hydrogel's mechanical strength, responsiveness, and other properties. The mechanical properties of DNA hydrogels can be characterized by rheological testing. The storage modulus (G’) of the material needs to be greater than the loss modulus (G’’) to prove to be a true gel. The mechanical properties of hydrogels are closely related to their internal structures. In general, the denser the crosslinking points are, the stronger the mechanical strength will be. Hydrogels with double-stranded structures exhibit superior mechanical strength compared to those primarily composed of single strands.

**Fig. 4:**
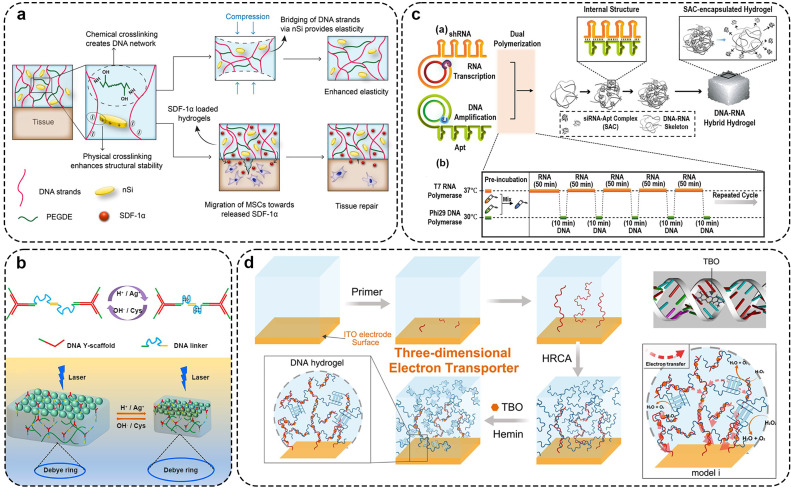
**Preparation of DNA hybrid hydrogel by multiple crosslinking.** (a) Synthesis of hydrogel for *in situ* tissue repair with physical and chemical crosslinkers. Adapted with permission from [123], copyright 2019, ACS Applied Materials & Interfaces. (b) Synthesis of responsive hydrogel by DNA nanostructure units and i-motif structure. Adapted with permission from [125], copyright 2022, Analytical Chemistry. (c) DNA−RNA hybrid hydrogel synthesis by double-enzyme polymerization process and physical entanglement. Adapted with permission from [73], copyright 2020, ACS Applied Materials & Interfaces. (d) Synthesis of hydrogel by RCA technology and electrostatic interaction. Adapted with permission from [126], copyright 2020, ACS Applied Materials & Interfaces.

The preparation of multicomponent DNA hydrogels can be achieved by the electrostatic interaction between positively charged materials. In a representative study, Zinchenko *et al*. reported a case of DNA-carbon nanotube hybrid hydrogel [121]. They first used ultrasound to crosslink multi-walled carbon nanotubes with DNA by electrostatic adsorption, then used EGDE to chemically crosslink with additional free DNA to form a gel at alkaline pH and high temperature. Similarly, Yan et al. used Pickering emulsion to create a high-pore DNA/CNT hydrogel capable of adsorbing the carcinogen PAH [122]. This study showed that CNTs can be used to change the porosity and adsorption properties of hydrogels. Paul et al. chemically crosslinked DNA with PEGDE to synthesize a DNA scaffold, then exploited the anisotropic charge distribution of 2D silicate nano-disks to create additional physical network crosslinking points through electrostatic interactions with the DNA scaffold. This approach enhanced the hydrogel's mechanical elasticity, resulting in a dual-crosslinked DNA composite hydrogel with both chemical and physical crosslinking points [123] (Fig. 4a). In addition, Liu et al. synthesized responsive dynamic double-crosslinked DNA hydrogels by using two types of covalent crosslinking agent [124]. First, a chemical crosslinking agent (Bis) creates an initial polymeric network by covalently linking two linear polymer chains in the gel. At the same time, ssDNA was covalently modified by free radical double bond polymerization to form a polymer network, which was used as a secondary crosslinking agent. Initially, ssDNA remained single-stranded until DNA bridges were added to the system and connected by hydrogen bonds, forming a set of secondary crosslinks. The overall volume of the hydrogel shrinks due to the compression of the inter-chain distance between adjacent linear polymers. This method, requiring only an external DNA (B sequence) for new crosslink formation, significantly lowers the cost of DNA-responsive hydrogels.

Photonic crystal (PhC) is a unique optical material formed by periodic arrangement of materials with different dielectric constants. Wu et al. combined the two-dimensional PhC array with DNA Y-scaffold and the linker units to design a two-dimensional PhC DN-DNA hydrogel [125]. The two-dimensional PhC array is selected as a signal conversion element to impart a debye diffraction signal to that hydrogel. The second crosslinked hydrogel network (PAM hydrogel) was introduced *in situ* into DNA supramolecular hydrogel to reduce the cost of DNA hydrogel and improve its mechanical properties. The hydrogel responds to a pH value or Ag^+^/Cys concentration and also has good selectivity and recyclability. It provides an advanced idea for constructing a novel multifunctional DNA hydrogel sensing platform (Fig. 4b). Lee et al. first prepared polymer shRNAs by step-by-step double-enzyme polymerization to hybridize with functional DNA inducers, and then made a DNA-RNA hybrid hydrogel by repeated hybridization and strand entanglement at multiple hybridization sites [73]. Furthermore, they encoded restriction enzyme reaction sites within the DNA-RNA hybrid hydrogel, enabling it to mimic microtubule structures under physiological conditions and sequentially release functional siRNA-aptamer complexes. This injectable and double-release DNA-RNA hydrogel has great potential in RNA therapy (Fig. 4c). Zhu et al. first initiated linear RCA on the electrode surface, followed by the activation of HRCA technology to form a DNA hydrogel network serving as a scaffold for electron transfer. Subsequently, toluidine blue O (TBO) and hermin were introduced as connecting nodes between branched DNA chains to stabilize the DNA network. Additionally, DNAzyme with intrinsic peroxidase-like activity were incorporated to create three-dimensional electron-transferring DNA hydrogels. (Fig. 4d) [126].

#### 3.2.5 Others

Schiff base reaction refers to the condensation of amines and active carbonyl groups, which is widely used for the crosslinking of hydrogels. For example, Basu et al. utilized the amino groups in DNA nucleotides to form reversible covalent imine bonds with the aldehyde groups in oxidized sodium alginate [127]. The covalent imine bond imparts self-healing and shear-thinning properties to the hydrogel. Besides, the nanomaterials and DNA can also be crosslinked by covalent interactions to prepare multicomponent DNA hydrogels. M. Niemeyer et al. coupled silicon dioxide nanoparticles (SiNP) and aminoalkyl-modified ssDNA primers through glutaraldehyde-based crosslinking to obtain primer-modified SiNP-ssDNA [128,129]. They then employed RCA technology for DNA amplification, resulting in a multi-component DNA hydrogel interwoven with DNA and SiNPs.

The multifunctional DNA hybrid hydrogel can be prepared by utilizing the property of the polymer itself. For example, Li et al. designed a supramolecular polypeptide DNA hydrogel by hybridization of linear DNA grafted on the polypeptide and the ‘sticky end’ of linear DNA double chain (linker) [130]. In an oscillating shear rheology test, the G’ of the hydrogel was 4845.5 ± 95.6 Pa (fixed strain 1%, frequency 1 Hz, 25℃) and the mechanical property of the hydrogel could be converted by the pH-sensitive property of the secondary structure of the polypeptide skeleton.

## 4 Advantages of DNA hydrogel in cartilage repair

### 4.1 Biocompatibility, biodegradability and bioabsorbability

The ideal hydrogel for cartilage repair cannot inhibit the normal biological activity of cells, interfere with or inhibit molecular signaling pathways, or cause inflammatory reactions [131,132]. For pure DNA hydrogels, the use of DNA, a macromolecule essential for the normal growth and development of most organisms, can circumvent immunogenicity issues. For DNA hybrid hydrogel, additional materials should also have good biocompatibility and biodegradability. In addition, the construction of cartilage organoids is a dynamic and continuous process. Therefore, DNA scaffolds for the construction of cartilage organoids should have an appropriate degradation rate. During the construction of cartilage organoids, DNA hydrogels can be gradually degraded by human DNases, leaving space for new cartilage tissues. The degradation products are nucleotides, which have no cytotoxicity and can be absorbed by cells to provide a nutritional basis for cartilage regeneration.

### 4.2 Excellent delivery capability

In the past decades, regenerative therapy based on mesenchymal stem cells (MSCs) has attracted more and more attention in the treatment of OA, because it can achieve therapeutic effects such as improving cartilage morphology and relieving pain and inflammation [133], [134], [135], [136], [137]. It has been reported that MSCs can supply differentiated progeny and secrete factors for repairing damage and modulating immunity, contributing to tissue regeneration [138], [139], [140]. Therefore, the delivery efficiency and long-term survival of MSCs are crucial for the treatment of OA. However, directly injecting mesenchymal stem cells into the knee joint can cause several issues. Cells may enter the systemic circulation, leading to cell loss. This results in a short retention time, which affects cell homing. There is also significant loss of stem cell microvesicles. Additionally, the adverse environment within OA impacts cell survival. These factors ultimately affect the effectiveness of therapy. Using hydrogel systems to deliver stem cells can prevent cell loss, improve stem cell utilization, provide lubrication, and extend the duration of effective action, thereby enhancing therapeutic effect.

**Fig. 5:**
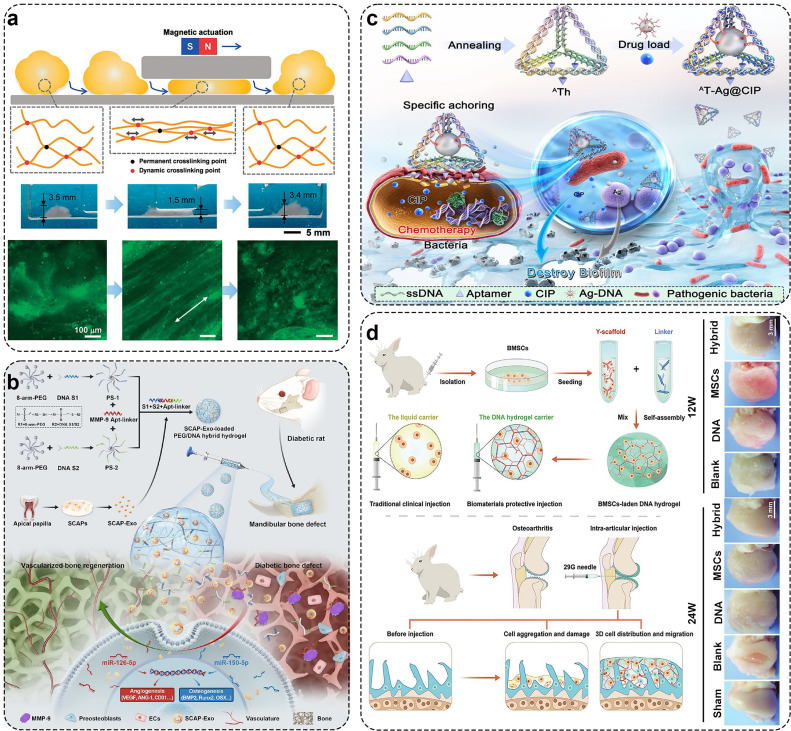
**Excellent delivery capability of DNA hydrogel.** (a) DNA hydrogel transports cells in a closed space. Adapted with permission from [143], copyright 2020, Angewandte Chemie-International Edition. (b) SCAP-Exo-loaded PEG/DNA hybrid hydrogel promote vascularized bone regeneration. Adapted with permission from [144], copyright 2022, ACS Applied Materials & Interfaces. (c) Double-bundle DNA tetrahedron targeted drug delivery. Adapted with permission from [145], copyright 2023, Aggregrate. (d) Injectable BMSCs-laden DNA supramolecular hydrogel treat cartilage injury. Adapted with permission from [147], copyright 2021,  Advanced Materials.

Yao et al. used RCA technology to amplify ultra-long DNA chains containing aptamer sequences of multiple stem cells to specifically anchor and capture stem cells [141]. The encapsulation of the stem cells was completed by forming a three-dimensional DNA hydrogel network through hybridization and complementary pairing between chains. Controlled release of stem cells was achieved as the DNA network degrades under nuclease activity. Additionally, numerous cases utilized DNA hydrogels as cell delivery systems, such as using DNA-assembled supramolecular hydrogel matrices to carry homologous neural stem cells for treating spinal cord injuries and achieving magnetically driven navigational motion to transport living cells within enclosed and unstructured spaces (Fig. 5a) [142,143]. Paul et al. reported the use of silicon-DNA hydrogels for *in situ* tissue repair by directed stem cell migration [123]. In addition, DNA hydrogels loaded with exosomes have been investigated to promote bone regeneration (Fig. 5b) [144]. Song et al. prepared the PEG/DNA hybrid hydrogel by using click chemistry and base-pairing reactions. The hydrogel has good response to matrix metalloprotein-9 (MMP-9) and can be changed in viscoelasticity by adding MMP-9. Besides, the DNA hydrogel has good injection performance and self-healing ability, and plays an important role in transporting exosomes to promote bone regeneration. Furthermore, numerous studies have explored the targeted transport and release of drugs leveraging the programmability of DNA. For example, Liao et al. designed a double-bundle DNA tetrahedron for targeted drug delivery (Fig. 5c) [145]. This dual-stranded DNA tetrahedron features aptamers that can bind to specific pathogens, carrying drugs within its cavity.

At present, DNA supramolecular hydrogels have been reported to transport bone marrow mesenchymal stem cells (BMSCs). Yang et al. reported the formation of a physically crosslinked hydrogel through hybridization and entanglement of ultralong DNA chains. The hydrogel was then modified with magnetic nanoparticles. This modification allowed for the specific capture, three-dimensional encapsulation, and enzyme-induced release of BMSCs [146]. The high programmability of the DNA network facilitated efficient cell capture and release, while its 3D porous structure and good biocompatibility ensured the high viability of the captured stem cells. Yu et al. designed a case of injectable DNA supramolecular hydrogel prepared by Y-type scaffolds and linkers (Fig. 5d) [147]. The hydrogel can effectively protect BMSCs from short-term shearing force and long-term joint friction *in vivo* and *in vitro*. The cell survival rate delivered by this DNA supramolecular hydrogel is as high as 99% (compared to 70% with traditional methods). Additionally, under high-friction conditions, the DNA supramolecular hydrogel effectively promotes cartilage formation, reduces osteophyte formation, and normalizes subchondral bone. Moreover, MSCs implanted in the DNA hydrogel exhibit higher activity and lower aggregation [148]. According to the latest research, Ding et al. used DNA supramolecular hydrogel to deliver metformin to treat OA [149]. This DNA hydrogel can not only greatly prolong the drug release time, but also play a role in reducing local inflammation. Therefore, the use of DNA hydrogel as a carrier to transport BMSCs has great prospects for the treatment of OA.

### 4.3 Intelligent response ability

**Fig. 6:**
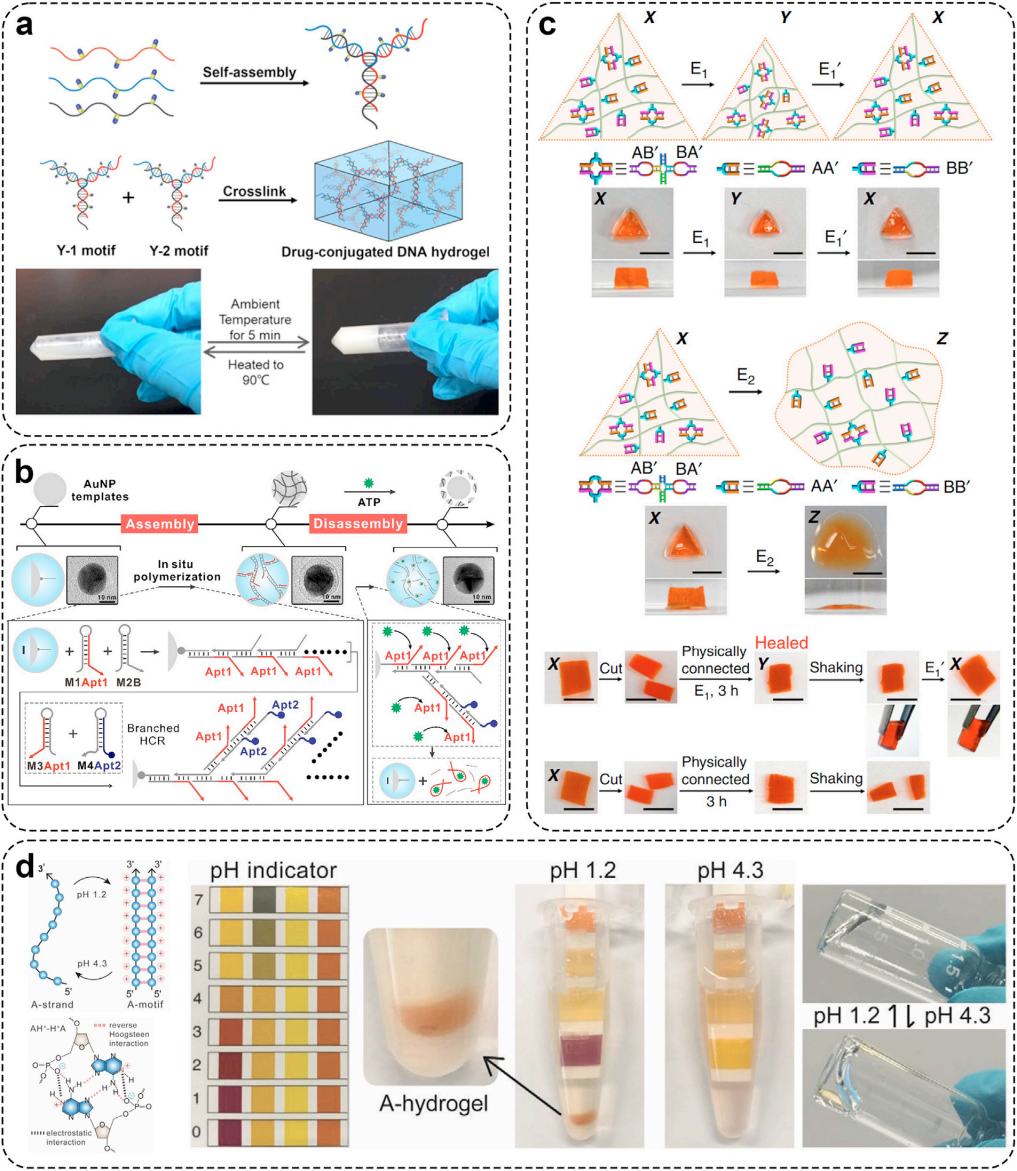
**Intelligent response ability of DNA hydrogel.** (a) The CPT-conjugated DNA hydrogel response to temperature. Adapted with permission from [62], copyright 2020, ACS Applied Materials & Interfaces. (b) The ATP-responsive spherical nucleic acidtemplated hydrogel. Adapted with permission from [157], copyright 2019, Journal of the American Chemical Society. (c) Visual imaging of the triggered CDN hydrogels’ stiffness changes and self-healing of the CDN hydrogels. Adapted with permission from [41], copyright 2019, Nature Communications. (d) PH-regulated sol-gel transformation. Adapted with permission from [159], copyright 2023, Small.

In addition to its specificity and affinity for target molecules, DNA has several practical advantages, such as biodegradability, biocompatibility, higher stability and simplicity in chemical modification. DNA hydrogel as an intelligent drug loading carrier shows unique advantages [150]. Dexamethasone (DEX) is an effective drug for the treatment of OA. Currently, there have been many reports about the intelligent release of DEX to treat diseases [151,152]. Ren et al. combined DEX with the prepared DNA/poly(lactic-co-glyco-licacid) hybrid hydrogel, facilitating the gradual release of DEX in ophthalmic cells and tissues. [153]. The ocular inflammation symptoms were significantly alleviated during the action of the hydrogel. Zhang et al. crosslinked chitosan with DNA molecules via electrostatic interactions to form injectable DNA hydrogels, which could carry DEX to achieve osteogenic differentiation [58]. Furthermore, there are many other examples of DNA hydrogel intelligent response release. As shown in Fig. 6a, Zhang et al. grafted camptothecin onto the backbone of phosphorothioate DNA, with two types of Y-shaped DNA building blocks being layered together to form an injectable drug-loaded hydrogel [62]. Due to enzymatic degradation, the hydrogel can gradually decompose into nanoparticles, which then penetrate the remaining tumor tissue and are efficiently absorbed by the cells. Furthermore, by introducing functional nucleic acids such as aptamers, accurate drug release into targeted tumor cells can be achieved. Zheng et al. utilized self-assembled DNA hydrogel as a soft scaffold to carry cytokine interleukin-10 (IL-10) to prolong the storage time of IL-10. Thereby achieving the effects of locally and continuously releasing the IL-10 at the defect part and promoting the reconstruction of diabetic alveolar bone [154]. Doxorubicin is a chemotherapeutic drug widely used in a variety of cancers. It can be used as a DNA intercalation drug that is embedded in the 5′-GC-3′ or 5′-CG-3′ of the DNA skeleton for enrichment. Many research teams have designed different drug delivery systems by combining Dox with DNA hydrogels based on this property [155,156]. Based on the prior research that intracellular ATP can stimulate the effective release of drugs within drug delivery systems, Pei et al. prepared a switch-engineered spherical nucleic acidtemplated hydrogel (SNAgel) with a DNA toehold switch, which was stimulus-responsive to ATP to achieve the release of the anticancer drug Dox (Fig. 6b) [157]. With Gold nanoparticles (AuNPs) as the core, an initiating DNA chain (I) was assembled onto it via Au-S bond to form a multivalent SNA with multiple reaction sites, which subsequently triggered the HCR reaction. Two DNA hairpin structures were designed, each containing two aptamer sequences. Apt1 is the ATP-specific toehold reaction switch, and Apt2 is an aptamer that can bind to PTK7, which is overexpressed in specific tumor cells. There are two kinds of HCR reactions, linear HCR and branched HCR. Au-I with linear structure triggered the linear polymerization of LP with M1Apt1 and M2B to obtain polymer. Subsequently, M2B initiated branched HCR with hairpin M3Apt1 and hairpin M4Apt2 to obtain SNAgel. The existence of Apt2d enables SNAgel to selectively enter specific tumor cells. ATP specifically binds to Apt1, which causes the change of toehold switch structure to destroy DNA double strand, and finally leads to the dissociation of SNAgel, thus realizing the rapid release of pre-embedded Dox to reach therapeutic concentration. Additionally, the hydrogel can also regulate the disassembly rate through the chain length of the aptamer toehold and selectively bind to tumor cells, thereby significantly enhancing the therapeutic effect and reducing the toxic and side effects. Singh *et al*. attached C-rich DNA through carbon sites and crosslinked the DNA by forming i-motif sequences to form hydrogels [158]. The drug can be embedded in DNA chains by Dox or encapsulated in multicomponent DNA hydrogels by electrostatic interaction with carbon sites. Hydrogels remain stable for a month under normal physiological pH and slowly releases Dox in an acidic pH microenvironment for over 10–11 days.

The mechanical properties of hydrogels will affect the transportation of nutrients, the distribution of nascent matrix, and the stability of structures during and after implantation. Chondrocyte differentiation is a dynamic process, Willner *et al*. designed a DNA constitutional dynamic network that imitates the function of natural network and can adapt, convert and reversibly convert in three different states [41]. The DNA hydrogel not only can show three different hardness characteristics but also can realize self-healing under the induction of enzyme (Fig. 6c). Using the deprotonation of A, Ying et al. designed a unique parallel A-motif double chain. The hydrogel composed of the A-motif double chains can be released by performing gel-to-solution conversion under strong acid conditions (Fig. 6d) [159]. This kind of DNA hydrogel has wide application prospect in protecting oral medicine from strong acid and digestive enzyme in bad gastric environment.

Utilizing this smart response ability, DNA hydrogels can offer a dynamic 3D environment for cell, conducive to investigating how cells grow and interact in constantly shifting conditions. Alternatively, by modulating the hydrogel's stiffness and pore structure, it is possible to affect cell behavior. Therefore, the intelligent response ability of DNA hydrogels demonstrates considerable potential for the cultivation of cartilage organoids. can provide a dynamically changing three-dimensional environment, aiding in the study of how cells grow and interact under varying conditions.

### 4.4 Excellent 3D culture scaffold

Presently, it has been reported that callus organoids are used to repair bone injuries, and trabecular bone organoid models are used to study local bone remodeling regulation [160], [161], [162]. Hence, constructing cartilage organoids as *in vitro* 3D models for cartilage regeneration or as transplant materials holds promise for offering new insights into cartilage tissue engineering. However, human cartilage tissue engineering is a complex process, as the differentiation of chondrocytes requires regulated signaling in an orderly manner both temporally and spatially [163]. Currently, it has been reported that DNA hydrogel had been used in bio-imaging and immobilization of three-dimensional cell culture to understand cell behavior and function in the three-dimensional environment [164,165]. In consequence, the construction of 3D network scaffold similar to cartilage ECM to support the expansion of chondrocytes is crucial for the *in vitro* formation of human cartilage organoids [131,^166^,167].

**Fig. 7:**
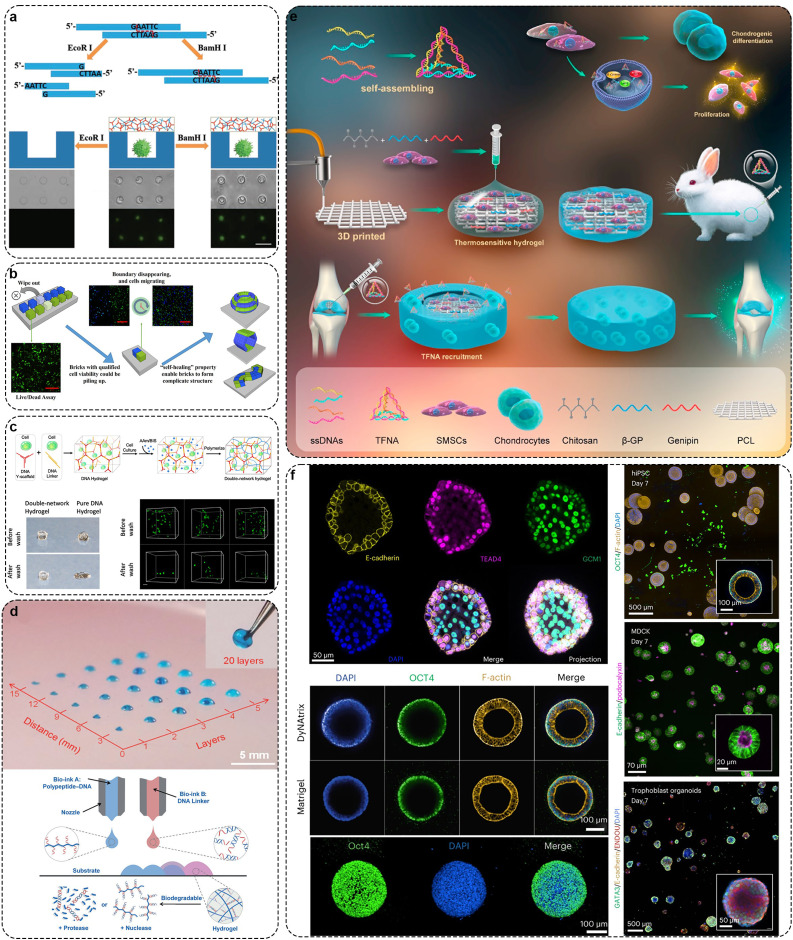
**DNA hydrogel can be used as an excellent 3D culture scaffold.** (a) The triggered DNA hydrogel release and envelop single cells. Adapted with permission from [168], copyright 2013, Advanced Materials. (b) DNA hydrogel brick for encapsulating different types of cells. Adapted with permission from [169], copyright 2017, ACS Applied Materials & Interfaces. (c) DNA−PAAm double-network hydrogel 3D culture cells. Adapted with permission from [165], copyright 2020, ACS Applied Materials & Interfaces. (d) 3D bioprinting polypeptide–chitosan-DNA hydrogel scaffold loaded with stem cells to treat cartilage injury. Adapted with permission from [175], copyright 2015, Angewandte Chemie-International Edition. (e) Schematic diagram of the design of 3D-printed DNA hybrid hydrogel. Adapted with permission from [179], copyright 2021, Biomaterials. (f) DNA hydrogel culture organoids. Adapted with permission from [180], copyright 2023, Nature Nanotechnology.

DNA hydrogels provide an elastic, mild ECM-like environment and conditions for the proliferation and migration of living cells, with a 3D network pore structure facilitating the transport of nutrients and the expulsion of cellular metabolic waste (Fig. 7a, b) [168,169]. Zuo *et al*. designed an aptamer-triggered C-HCR to form a porous DNA hydrogel for direct capture and release of circulating tumor cells (CTCs) [170]. The aptamer sequence on the atcHCR initiation chain can specifically recognize the epithelial cell adhesion molecule on the surface of CTCs, initiating branch migration that triggers subsequent atcHCR. Confocal microscopy images confirm that the porous DNA hydrogel prepared by this method can capture live CTC cells *in situ*, with minimal damage to the captured cells. Additionally, the hydrogel is designed with ATP-responsive sequences. The presence of ATP in the system can cause the hydrogel to dissolve from the cell surface, releasing the cells. Moreover, Yang et al. developed a supramolecular assembly technique using DNA and upconversion nanoparticles, driven by electrostatic and coordination interactions, for synthesizing large-scale hydrogels rapidly [171]. This approach also allowed for selective encapsulation of target cells and non-specific encapsulation of non-target cells through careful DNA sequence design. Cao et al. constructed a stable 3D cell culture system for cell immobilization and imaging by using covalent polyacrylamide as the second network in a DNA hydrogel which composed of Y-scaffolds and linkers (Fig. 7c) [165]. Compared to single-component hydrogels, dual-network hydrogels exhibit improved mechanical properties under tension and shear. Cells encapsulated within them are well-distributed in three dimensions. Shao et al. constructed a self-assembled DNA hydrogel using dendritic DNA and linkers [172]. This hydrogel can interfere with cell growth by loading various exogenous substances. Within this DNA hydrogel, both somatic and tumor cells exhibit high proliferation. Liu et al. designed a self-assembled L-DNA hydrogel composed of Y-scaffolds and linkers. L-DNA hydrogels possess enhanced biostability to support long-term cell culture [173]. In addition, reports suggest that peptide-functionalized DNA hydrogel scaffolds can enhance the growth and differentiation of neuroblastoma cells. [174].

The bioorthogonal of DNA hydrogels can be easily applied by advanced techniques such as bio-printing, bio-ink and creating three-dimensional shapes (Fig. 7d) [175]. Wang et al. developed a dynamic DNA hydrogel supported by black phosphorus nanosheets that could be used for 3D printing [176,177]. The black phosphorus nanosheets enhanced the mechanical strength of the dynamic self-healing hydrogel. Besides, the noncovalent interactions between the growth factor and the black phosphorus nanosheet can continuously release the growth factor to achieve enhanced angiogenesis and bone regeneration. Acrylamide hydrogels, modified with DNA via ligand affinity polymerization, demonstrate nanoscale precision in programmable signals. These signals induce and control interactions with the cell membrane, showing multivalent ligand affinity towards cell adhesion, differentiation, and proliferation [178]. Tetrahedral framework nucleic acid (TFNA), composed of four self-assembling single-stranded DNAs into a stable tetrahedral structure, can enter cells to modulate various signaling pathways. When injected into the joint cavity of animal models, it significantly enhances the proliferation and differentiation of MSCs, facilitating cartilage regeneration. Hao *et al*. injected a chitosan hydrogel mixed with MSCs into 3D-printed poly (ε-caprolactone) (PCL) scaffolds. Then, TFNA was injected into the joint cavity, where it physically interacted with the chitosan hydrogel to achieve targeted recruitment. The chitosan hydrogel /3D printed PCL mixed scaffold containing MSCs can enhance the cell proliferation and cartilage regeneration after the addition of TFNA, and has a promising future in cartilage tissue engineering (Fig. 7e) [179]. At present, there have been studies on organoid culture based on DNA hydrogel [180]. This kind of DNA hydrogel combines acrylic-co-acrylic acid backbone with complex DNA library. These DNA library crosslinking agents can adjust the stress relaxation of hydrogels only by changing a few bases on the crosslinking agent sequence. The authors used this DNA hydrogel to culture organoids based on human induced pluripotent stem cells, Madin–Darby canine kidney cells and human trophoblast stem cells. Notably, trophoblast organoids can be cultured for up to 3 weeks under the condition of serum-free medium supported by DNA hydrogel (Fig. 7f). All in all, DNA hydrogel has enormous potential as a 3D scaffold for the culture of cartilage organoids.

## 5 Research progress in cartilage organoids

The joint surfaces of synovial joints are enveloped by a layer of smooth hyaline cartilage, serving as a biomechanical "cushion" that mitigates stress during joint articulation and safeguards the underlying bone structure. Within this specialized tissue, chondrocytes comprise merely 2%−5% of the total volume, embedded within an ECM predominantly constituted of water, Type II collagen (Col II), and glycoproteins. Col II imparts tensile strength and preserves the cartilage's morphological integrity, while proteoglycans substantially contribute to the compressive resilience, modulating osmotic balance and facilitating the flux of water, thereby regulating the cartilage's internal and external pressure dynamics [181,182]. Ideal cartilage organoids should replicate this intricate 3D architecture, featuring an ECM replete with Col II and proteoglycans, and encapsulating viable chondrocytes. Such organoids are envisioned to not only mirror the mechanical properties of native cartilage but also possess the potential for seamless integration into host tissues, thus playing an instrumental role in the restoration and rejuvenation of compromised cartilage [183], [184], [185].

At present, the methods for constructing organoids can be broadly classified into two categories: scaffold-free self-organization and biomaterial co-cultivation. Each of these methods has its own set of advantages and disadvantages. Scaffold-free self-organization in tissue engineering offers a process that parallels natural organ development by allowing cells to self-assemble into complex structures, offering simplicity and a reduced risk of contamination due to the lack of foreign materials. However, this approach faces challenges such as limited control over the resulting organoid's size and structure, inherent variability between batches, and difficulties in scaling the process for larger production. In contrast, biomaterial co-cultivation provides distinct advantages by using scaffolds, such as hydrogels, to exert precise control over organoid architecture, ensuring uniformity and predictability. Biomaterials also deliver critical structural support, especially for organoids that simulate solid organs, and offer versatility to customize the microenvironment to suit specific types of organoids [186].

**Fig. 8:**
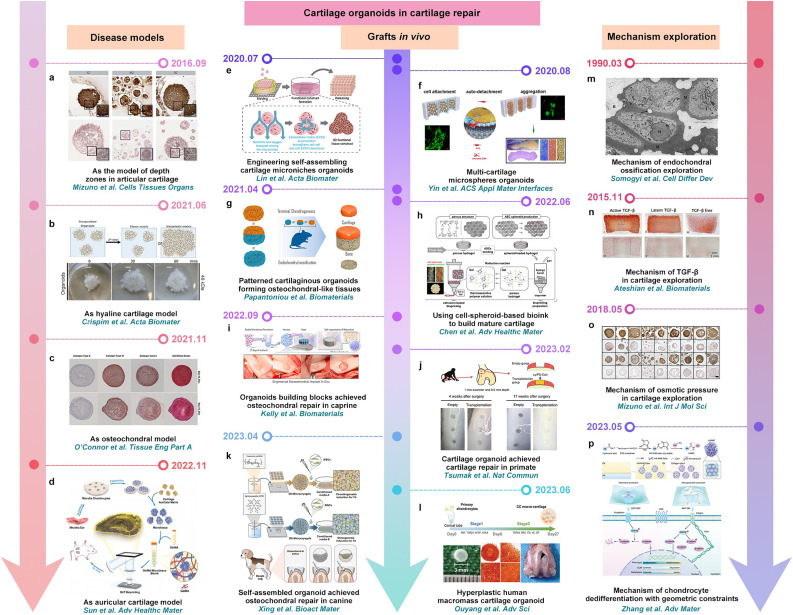
**Recent developments of cartilage organoids in cartilage repair.** (a) Adapted with permission from [187], copyright 2016, Cells Tissues Organs. (b) Adapted with permission from [21], copyright 2021, Acta Biomaterialia. (c) Adapted with permission from [188], copyright 2021, Tissue Engineering Part A. (d) Adapted with permission from [189], copyright 2022, Advanced Healthcare Materials. (e) Adapted with permission from [190], copyright 2020, Acta Biomaterialia. (f) Adapted with permission from [191], copyright 2020, ACS Applied Materials & Interfaces. (g) Adapted with permission from [192], copyright 2021, Biomaterials. (h) Adapted with permission from [193], copyright 2022, Advanced Healthcare Materials. (i) Adapted with permission from [194], copyright 2022, Biomaterials. (j) Adapted with permission from [23], copyright 2023, Nature communications. (k) Adapted with permission from [195], copyright 2023, Bioactive Materials. (l) Adapted with permission from [196], copyright 2023, Advanced Science. (m) Adapted with permission from [197], copyright 1990, Cell differentiation and development. (n) Adapted with permission from [198], copyright 2016, Biomaterials. (o) Adapted with permission from [199], copyright 2018, International Journal of Molecular Sciences. (p) Adapted with permission from [200], copyright 2023, Advanced Materials.

Currently, numerous studies have been conducted on the construction of cartilage organoids for cartilage repair. Initially, from the perspective of disease models, scholars have developed comprehensive cartilage models including hyaline, osteochondral, and auricular constructs, pivotal for emulating disease progression and assessing therapeutic strategies [21,[187], [188], [189] (Fig. 8a-d). Focusing on grafting applications, researchers like Lin et al. and Yin et al. have exploited self-assembling materials to stack cartilage microspheres, facilitating the generation of large-volume organoids [190,191] (Fig. 8e, f). Papantoniou et al. engineered layered osteochondral organoids by integrating cartilage microtissues from iPSC-derived chondrocytes with callus organoids from hPDCs [192] (Fig. 8g). Chen et al. introduced a poly(N-isopropylacrylamide)-based hydrogel for bioink creation, streamlining the fabrication of custom-shaped mature cartilage, while Kelly et al. team used 3D printing to orchestrate microtissue assembly for joint cartilage restoration [193,194] (Fig. 8h, i). Notably, organoids crafted by Tsumaki et al. and Xing et al. have shown efficacy in cartilage repair within primate and canine models [23,195] (Fig. 8j, k). Furthermore, Ouyang et al. cultivated sizable macromass cartilage organoids to bridge cartilage defects [196] (Fig. 8l). Lastly, cartilage organoids have been instrumental in unraveling the mechanisms of endochondral ossification, TGF-β's role in cartilage biology, the significance of osmotic pressure in cartilage maintenance, and the impact of geometric constraints on chondrocyte dedifferentiation [197], [198], [199], [200] (Fig. 8m-p). In summary, researches on constructing cartilage organoids have provided multifaceted insights and prospective applications for cartilage repair.

## 6 Conclusion

DNA hydrogels, recognized for their programmability, modifiability, predictable secondary structures, biocompatibility, and biodegradability, are considered superior substrates for fabricating tissue engineering scaffolds. This paper reviews the recent advancements in the use of DNA hydrogels for tissue engineering, focusing on the preparation, functional modification, and advantages of DNA hydrogels. Extensive researches demonstrates that DNA hydrogels possess excellent biocompatibility, biodegradability, desirable smart responsiveness, and exceptional cell loading and delivery capabilities, making them ideal 3D culture scaffolds. Thus, DNA hydrogels hold significant potential in cartilage regeneration, repair, and organoid construction. Current researches on cartilage organoids in cartilage repair are focused on creating disease models, grafts, and exploring cartilage-related mechanisms. The use of DNA hydrogels in constructing cartilage organoids may provide new strategies for cartilage defect treatment.

## 7 Prospect

**Fig. 9:**
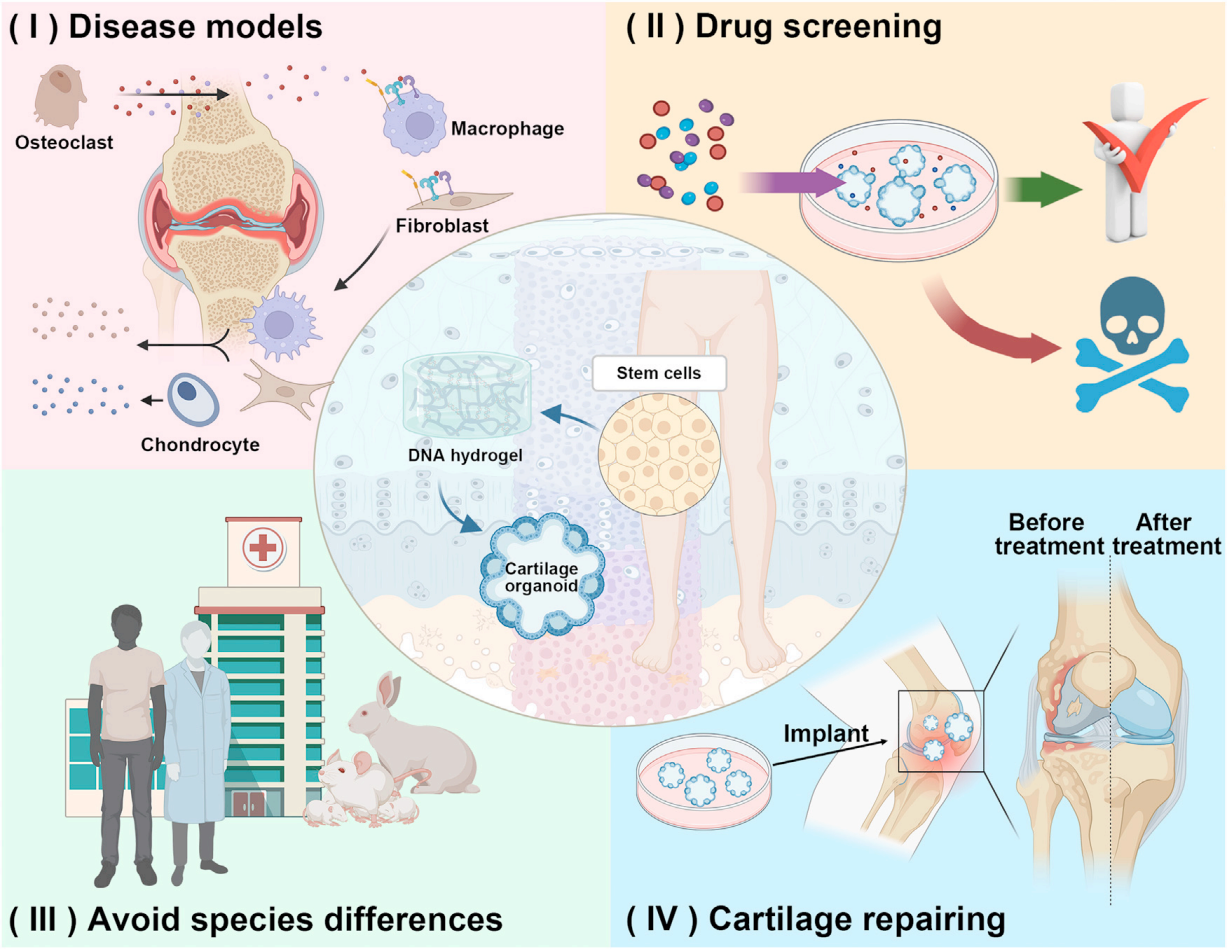
**Prospect of constructing cartilage organoids with DNA hydrogel.**. (Ⅰ) As *in vitro* disease models and defect models. (Ⅱ) Using cartilage organoids to model disease for drug screening. (Ⅲ) Using cartilage organoids instead of animals. (Ⅳ) As an implant for cartilage repair.

While several reports exist on using DNA hydrogels for cartilage repair with positive outcomes in both *in vitro* and *in vivo* experiments, focusing on mesenchymal stem cell loading and drug release, our team has pioneered the integration of DNA with silk fibroin to create a dual-network DNA-silk fibroin hydrogel with controllable surface stiffness for directing stem cell differentiation into cartilage. Moreover, the construction of cartilage organoid precursors using these novel DNA-silk fibroin hydrogels has markedly enhanced cartilage regeneration [201]. This novel DNA-SF hydrogel significantly enhances cartilage regeneration by constructing cartilage organoid precursors [202]. Leveraging a biomimetic multilayer scaffold stimulates specific zonation of native cartilage tissue, promoting ideal cartilage and bone formation, representing an advanced strategy in articular cartilage regeneration [138,203]. Using the programmability of DNA hydrogel to construct biomimetic multilayer scaffold is an ideal scheme. In the future, researchers should advance the use of DNA hydrogel as 3D culture scaffold to construct cartilage organoids, which is expected to break the bottleneck of cartilage repair research, including (1) Employing cartilage organoids as *in vitro* disease and defect models facilitates the exploration of disease progression mechanisms, cartilage repair processes, and drug action mechanisms. (2) Using cartilage organoids to model disease for drug screening. (3) Using cartilage organoids instead of animals to evaluate the regeneration and repair function and mechanism of biomaterials, which avoids the ethical problems and clinical translation difficulties caused by the species differences [185]. (4) As an implant for cartilage repair, replacing autologous cartilage transplantation. We believe that DNA hydrogels are ideal materials for the construction of cartilage organoids and can provide new methods for cartilage regeneration and repair (Fig. 9).

At present, Matrigel is the most widely used hydrogel to construct organoids, which is a commercial hydrogel prepared by the ECM of Engelbreth-Holm-Swarm mouse sargoma cells. Although Matrigel has been maturely used for organoid cultures, including the cultivation of ultimate and cortical tissues, gastric organoids, pancreas organoids, colonic organoids, liver organoids, and intestinal organoids, it still has several drawbacks [204], [205], [206], [207], [208], [209]. For example: (1) Because of the differences between different batches of animals, the reproducibility of products and experimental results is poor. (2) The components contained in Matrigel are not clear. The culture scaffold with clear components is a better alternative. (3) The biomechanical properties of Matrigel in different batches are also different. (4) Matrigel is difficult to be customized according to the culture needs of organoids. On the contrary, the preparation of DNA hydrogel does not depend on animals, so there is no batch difference. Additionally, Matrigel is not readily customizable to meet the specific culture needs of particular organs. DNA hydrogels, produced through artificial amplification techniques, do not suffer from batch-to-batch variability. In addition, DNA hydrogels are free from animal-derived materials, thus lowering the risks of immunogenicity and pathogen transmission. Moreover, DNA hydrogels offer greater transparency, facilitating cellular imaging with microscopy. Furthermore, DNA hydrogels can leverage their intrinsic base-pairing complementarity or be utilized in conjunction with photo-crosslinking suitable for 3D bioprinting. Furthermore, by editing specific sequences, it's possible to regulate mechanical properties or release drugs over time and space, adapting to the varying environmental requirements of organoids at different stages of cultivation. However, the potential of DNA hydrogels as 3D culture scaffolds for cartilage organoids remains underexplored. For instance: (1) In-depth involvement of hydrogel dynamics: The dynamics of hydrogels directly impact cell behavior, differentiation, and ultimately, the functionality of the tissue. Current preparation methods for DNA hydrogels are primarily focused on achieving functionality in a rather qualitative manner. Research on hydrogel dynamics can provide references for the optimization and practical application of hydrogels. Studies on the kinetics of sol-gel transitions, the forces generated, and transition reversibility offer a theoretical basis. This basis supports the development of shape-memory hydrogels and controlled release mechanisms for hydrogels combined with bioactive substances, aiming to mimic the biomechanical environment of natural cartilage tissue. (2) Precise control of physicochemical parameters: Currently, the mechanical properties of most DNA-based hydrogels must be improved for use in cartilage tissue engineering. Their tensile strength should match that of cartilage tissue. Constructing cartilage organoids is a dynamic process. Precise control over DNA hydrogels' physicochemical parameters can facilitate transitions between low, medium, and high strength states, catering to diverse functional requirements. Moreover, there has been limited study on the stability and durability of the original functions of DNA hydrogels during reconstruction, stretching, and displacement processes after several driving cycles, especially after introducing concepts such as self-healing and stretchability into DNA hydrogels. Therefore, precise control over the physicochemical parameters of DNA hydrogels is particularly crucial in the research of such novel materials.

In summary, DNA hydrogels have broad research prospects in the field of cartilage repair. With the advancement of cartilage repair, we proposed to use the programmability of DNA to develop an adaptively adjusted intelligent DNA hydrogel to ensure rapid cartilage repair. We believe that DNA hydrogels have broad application prospects in cartilage repair and cartilage organoid construction.

## Declaration of competing interest

The authors declare that they have no conflicts of interest in this work.
